# Left inferior frontal gyrus is critical for response inhibition

**DOI:** 10.1186/1471-2202-9-102

**Published:** 2008-10-21

**Authors:** Diane Swick, Victoria Ashley, And U Turken

**Affiliations:** 1 Research Service, Veterans Affairs Northern California Health Care System, Martinez, CA 94553, USA; 2 Department of Neurology, University of California, Davis, Martinez, CA 94553, USA

## Abstract

**Background:**

Lesion studies in human and non-human primates have linked several different regions of prefrontal cortex (PFC) with the ability to inhibit inappropriate motor responses. However, recent functional neuroimaging studies have specifically implicated right inferior PFC in response inhibition. Right frontal dominance for inhibitory motor control has become a commonly accepted view, although support for this position has not been consistent. Particularly conspicuous is the lack of data on the importance of the homologous region in the left hemisphere. To investigate whether the left inferior frontal gyrus (IFG) is critical for response inhibition, we used neuropsychological methodology with carefully characterized brain lesions in neurological patients.

**Results:**

Twelve individuals with damage in the left IFG and the insula were tested in a Go/NoGo response inhibition task. In alternating blocks, the difficulty of response inhibition was easy (50% NoGo trials) or hard (10% NoGo trials). Controls showed the predicted pattern of faster reaction times and more false alarm errors in the hard condition. Left IFG patients had higher error rates than controls in both conditions, but were more impaired in the hard condition, when a greater degree of inhibitory control was required. In contrast, a patient control group with orbitofrontal cortex lesions showed intact performance.

**Conclusion:**

Recent neuroimaging studies have focused on a highly specific association between right IFG and inhibitory control. The present results indicate that the integrity of *left *IFG is also critical for successful implementation of inhibitory control over motor responses. Our findings demonstrate the importance of obtaining converging evidence from multiple methodologies in cognitive neuroscience.

## Background

The ability to inhibit inappropriate responses is one of the key functions attributed to the frontal lobes [1] and a major component of "executive control" functions [2]. The Go/NoGo task, in which a motor response is given to one stimulus class and withheld to another, has been used extensively to assess inhibition in both animals and humans [3]. A standard model of the Go/NoGo task holds that prefrontal regions of the brain are responsible for inhibiting responses to inappropriate stimuli, signaling the motor system to override an automatic tendency to respond. This can be viewed in the framework of top-down control of behavior [4], in which:

"The PFC [prefrontal cortex] is critical in situations when the mappings between sensory inputs, thoughts, and actions either are weakly established relative to other existing ones or are rapidly changing. This is when we need to use the 'rules of the game,' internal representations of goals and the means to achieve them" (p. 168, Miller & Cohen, 2001).

Identifying the frontal areas specifically associated with inhibitory control has been a topic of considerable interest. However, there is still a lack of consensus about which specific subregions in prefrontal cortex (PFC) are involved in response inhibition. Lesion and single-unit recording studies in primates have implicated lateral orbital PFC, sulcus principalis, and periarcuate regions in correct withholding of responses to NoGo stimuli [5-7]. In contrast, the human lesion literature has reported response inhibition deficits after damage to dorsomedial frontal areas [8-12]. Another investigation showed higher rates of NoGo errors in patients with lesions in basal and lateral PFC of either hemisphere, but this deficit was attributed to additional damage in the left caudate [13].

Several neuroimaging papers have argued that right hemisphere regions in PFC, particularly dorsolateral PFC and inferior frontal gyrus (IFG), are predominant for inhibitory control [14-18]. However, most of these studies, as well as others [19-26] did observe activations in bilateral dorso- and ventrolateral PFC, as well as medial PFC. Precise neuroanatomical analysis in patients with frontal lobe damage can reveal which of these areas are *necessary *for response inhibition. In a recent neuropsychological study, lesions of right IFG were associated with impairments in Stop-Signal inhibition [27]. In the Stop-Signal task, subjects always respond to go signals unless they are followed by a stop signal, which occurs on 25% of the trials at varying intervals after the go signal [28]. Patients with lesions in right IFG, but not other PFC regions, required longer to suppress a pre-planned response [27]. The authors concluded that the right IFG is uniquely associated with motor inhibition. However, divergent neuropsychological results were obtained in three other studies that did not observe Stop-Signal or Go/NoGo deficits in patients with right IFG damage [11,12,29]. Instead, these authors found impairments in patients with lesions in either left [12] or right superior medial PFC [11]. Therefore, the question of whether there is a strict parcellation of frontal lobe regions, with a specific role for RIFG in inhibitory control, still remains unanswered.

The literature on left IFG function motivates a specific test of its involvement in inhibitory control. This parallel area of research has examined the role of the posterior left inferior frontal gyrus (LIFG), or Broca's area, in executive functions involving the contribution of inhibitory control processes, such as semantic selection [30] and the resolution of proactive interference in working memory [31]. In the former case, patients with lesions in LIFG, but not other lateral PFC areas, made more errors on a verb generation task for nouns that had many possible responses (e.g., cat), but not for nouns that had few possible responses (e.g., scissors). In the latter case, a patient with a large LIFG lesion was impaired at inhibiting stimuli that were no longer relevant on the current trial of an item recognition task. Thus, a unified hypothesis of LIFG function might encompass the general theme of restraining alternatives in a given context, whether that context includes motor, semantic, mnemonic, or linguistic alternatives.

An interesting study on the developmental time course of prefrontal regions provides evidence for LIFG involvement in inhibitory control. Early in development, inhibitory control appears to be associated with LIFG regions, and the prominence of right IFG emerges only later in life [23]. Other lines of research using the Stroop color-word task (e.g., [32]), which shares in common with the Go/NoGo task the requirement to override automatic but task-inappropriate responses [33], also suggest that LIFG plays an important role in resolving conflicts that arise from incompatible representations [34]. Finally, anatomical studies have not established the pathways that would account for an exclusive RIFG involvement in response inhibition. Presumably, the right IFG would acquire its role in inhibitory control by virtue of its connections to the motor system [35]. The way in which the left IFG is interconnected with the basal ganglia, medial frontal cortex, and other components of the motor system is not known to be any different from the right IFG. These observations make it less likely that the left IFG should play no role whatsoever in response inhibition.

Because of the inconsistent support for right hemisphere dominance in inhibitory control in the neuroimaging and neuropsychology literatures and inadequate sampling of patients with LIFG lesions, additional neuropsychological studies are important for determining whether additional PFC regions are critical for response inhibition. The current experiment tested patients with lesions in the left inferior frontal gyrus (LIFG), and patients with orbitofrontal cortex (OFC) damage as a brain lesion control group. To manipulate the prepotency of responding, and hence the need for inhibitory control, the probability of NoGo stimuli alternated between 50% and 10%. Since the original support for a strong RIFG contribution to motor response inhibition came from the functional neuroimaging literature, which itself has produced varying findings, we have also consulted the BrainMap database [36] and other published papers in order to verify bilateral IFG involvement in neuroimaging investigations of inhibitory control. A quantitative meta-analysis of the relevant functional imaging studies was carried out using the activation likelihood estimation (ALE) method [37]. Given the observation of bilateral IFG activations in functional imaging studies of inhibitory control, the anatomical interconnections of the left and right inferior frontal gyri, and the known involvement of LIFG in response conflicts, the major question posed by the current study is whether the left IFG is crucial for accurate performance in the Go/NoGo task.

## Methods

### Subjects

**Figure 1:**
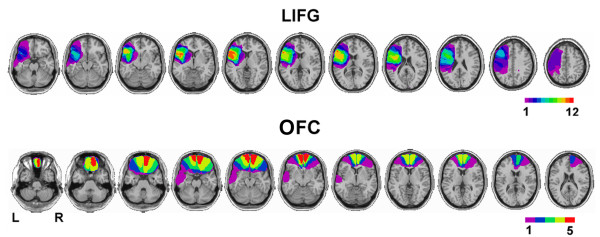
**Lesion Reconstructions**. Lesion reconstructions for the patient groups are shown as overlays onto sequential axial templates derived from the MNI brain. The left side of the brain is on the left side of the scans. The color scale bars depict the number of patients with lesion overlap in a given area. (a) Lesion overlap in patients with damage to the posterior left inferior frontal gyrus (LIFG) and insula. (b) Lesion overlap in patients with orbitofrontal cortex (OFC) damage (all bilateral).

Participants were 17 patients with lesions in the frontal lobes (mean age 57.2 yrs) and 16 age-matched controls (mean 58.0, range 41–72 yrs). Twelve LIFG patients were selected for single focal lesion visible on CT or MRI scans, caused by infarction in the precentral branch of the middle cerebral artery. These lesions were centered in Brodmann areas 6, 44, 45, and the insula, but damage included the portions of areas 9, 46, and 47 in some individuals and extended to the temporal tip in others. The mean age of the LIFG patient group was 58.6 yrs (range 42–71), and the mean time post-injury was 5.8 yrs. The OFC group consisted of five patients with bilateral ventromedial PFC lesions due to traumatic brain injury (TBI). Brodmann areas affected in these individuals included areas 11, 10, and extending posteriorly to BA 25 and laterally to anterior 45, 46, 47, in some (left temporal pole in one, right BA 9 in another). The mean age of the OFC group was 55.0 yrs (range 40–66), and the mean time post-injury was 20.0 yrs. Lesions were transcribed onto corresponding axial templates (the Montreal Neurological Institute (MNI) brain cut at a steeper slice angle) using MRIcro software [38]. Separate lesion overlays were obtained for each group (Fig. 1). The lesion reconstructions for individual patients are illustrated in Additional file 1 (LIFG) and Additional file 2 (OFC).

Patients with lacunar infarcts, white matter hyperintensities, significant medical complications, psychiatric disturbances, substance abuse, multiple neurological events or dementia were excluded. All subjects were right handed and were matched (approximately) for education level (LIFG 16.1 yrs; OFC 12.8 yrs; Controls 13.3 yrs). English was the primary language for all participants. The subjects were paid for their participation and signed informed consent statements approved by the Institutional Review Boards of the VA Northern California Health Care System and the University of California, Davis. All procedures were in compliance with the Declaration of Helsinki .

### Go-NoGo Task

Stimuli consisted of letters of the alphabet, printed in a large black font on a white background. Single uppercase letters were rapidly and serially presented at the center of a computer screen for 200 msec duration once every 1500 msec. Subjects were instructed to respond as quickly as possible to every letter except for "X" by pressing a button on the keyboard with the index finger of the right (dominant) hand. Because of right-sided hemiplegia or hemiparesis, 7 LIFG patients used the index finger of the left hand. In four alternate blocks, the proportion of "Go" to "NoGo" trials alternated between 50/50 and 90/10. There were 140 trials per block, with short rest breaks between each block. A short practice set of 30 trials (15 Go and 15 NoGo, randomly intermixed) preceded the experimental trials.

### Data Analysis

Error data were characterized as missed responses to Go stimuli and false alarm responses to NoGo stimuli. Reaction time (RT) data were sorted into correct hits to Go stimuli and incorrect false alarms to NoGo stimuli. Statistical analyses were carried out using repeated measures analyses of variance (ANOVAs) with factors of group (as described below) and probability of NoGo stimuli (50% vs. 10%). Planned comparisons (contrasts) or post-hoc tests (Fisher's Protected LSD) were used to further describe significant effects.

The patients (12 LIFG, mean age = 58.6 yrs; 5 OFC, mean = 54.0 yrs) were compared to an age-matched group of 16 controls (mean = 59.7 yrs). The OFC patients were also compared to a group of 8 controls more closely matched in age (mean = 54.6 yrs).

### Activation Likelihood Estimation

**Figure 2:**
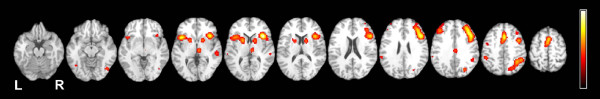
**Meta-analysis of Neuroimaging Studies**. Activation likelihood estimation (ALE) map showing significant inhibition-related activations overlaid on the International Consortium for Brain Mapping (ICBM) single subject template. The left side of the brain is on the left side of the scan.

Activation likelihood estimation (ALE) is a quantitative meta-analysis method [37] that can be used to infer function-location relationships from the functional neuroimaging literature. BrainMap is a searchable online database created and developed at the Research Imaging Center of the University of Texas Health Science Center San Antonio. At the time of this writing, the Sleuth program identified 46 papers reporting activations in Go/NoGo and Stop-Signal inhibition tasks, and 25 of these were included in the meta-analysis. Studies that were not conducted in young control subjects or that did not use manual responses were excluded. In addition, 14 more eligible papers (not included in the BrainMap database) were found through PubMed searches and entered into the meta-analysis. Since activation foci in BrainMap are specified using Talairach coordinates, the GingerALE program was used to make appropriate conversions from MNI to Talairach space using the icbm2tal transform [39] for these additional papers. Table 1 shows the list of studies that were included in the analysis and the number of activation foci for each (see Additional file 3 for full citations). The Talairach coordinates of all inhibitory control-related activations were used to estimate voxel-wise activation likelihoods. A FWHM (full-width half-maximum) of 12, a false discovery rate threshold of p < 0.01, and a cluster extent threshold of 100 mm^3 ^were applied to the ALE map. The resulting map identified the regions of activation common to all studies comprising the meta-analysis (Fig. 2).

**Table 1:** Studies Included in the Meta-Analysis

First Author	Year	Foci (n)
Aron	2006	35
Aron	2007	38
Asahi	2004	11
Bellgrove	2004	19
Braver	2001	19
Chikazoe	2008	104
deZubicaray	2000	26
Fassbender	2004	8
Garavan	1999	14
Garavan	2002	16
Garavan	2003	19
Hester	2004	21
Horn	2003	14
Kaladjian	2007	11
Karch	2008	13
Kawashima	1996	39
Kelly	2004	30
Kiehl	2000	8
Konishi	1998	19
Konishi	1999	1
Langenecker	2007	8
Laurens	2005	12
Leung	2007	7
Li	2006	3
Liddle	2001	42
Maguire	2003	16
Maltby	2005	5
Menon	2001	13
Mobbs	2007	4
Mostofsky	2003	6
Nakata	2008	33
Roth	2007	13
Rubia	2001	30
Rubia	2006	11
Vink	2005	8
Wager	2005	25
Watanabe	2002	9
Xue	2008	13
Zheng	2008	20

List of studies, including the number (n) of activation foci entering into the activation likelihood estimation (ALE) meta-analysis.

## Results

### Accuracy

ANOVAs were performed for errors of omission on Go trials (misses) and errors of commission on NoGo trials (false alarms) with factors of NoGo probability (50%, 10%) and group (controls, LIFG, OFC). In general, the rate of misses was very low (less than 1%) and not affected by the probability of NoGo trials (p > .9) or by group (p > .4). The percentage of missed responses for the 50% and 10% probability conditions was 0.27 and 0.35, respectively, for controls; 0.66 and 0.73 for LIFG; and 0.14 and 0.16 for OFC.

**Figure 3:**
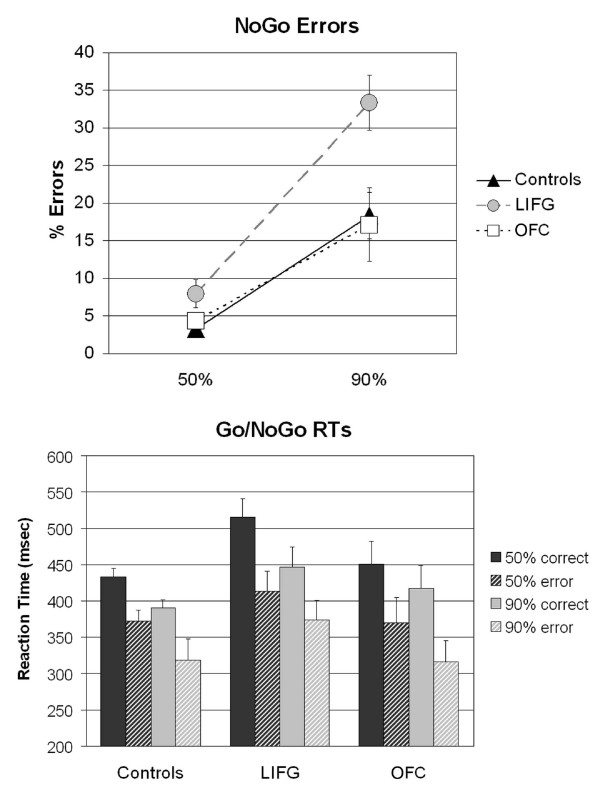
**Behavioral Data**. Error data (percentage of NoGo errors) and reaction time data (msec) from patient groups and age-matched controls. The error bars depict standard errors.

Conversely, NoGo errors were significantly affected by group [F(2,30) = 6.75, p < .005], NoGo probability [F(1,30) = 74.62, p < .0001], and a significant interaction between the two [F(2,30) = 4.94, p < .05]. Follow-up analyses compared the patient groups to their respective control groups. LIFG patients made more false alarm errors than controls for both the 50% [F(1,26) = 6.78, p < .05] and the 10% [F(1,26) = 11.13, p < .005] probability conditions (Fig. 3, top). In addition, the failure to inhibit inappropriate responses was more pronounced for LIFG patients in the 10% NoGo condition, in which responding was prepotent [F(1,26) = 8.33, p < .01]. This impairment pattern was not associated with contralesional motor responses only: there was no difference in false alarm rates between the LIFG patients who responded with the right hand and those who responded with the left hand (p = .27), nor was there an interaction with probability (p = .46).

In contrast, patients with orbitofrontal damage performed as well as controls. The ANOVA comparing the OFC patients to their age-matched control group found neither a main effect of group nor an interaction (p's > .5; see Fig 3). Furthermore, the OFC patients were more accurate than the LIFG group [F(1,15) = 4.80, p < .05], and again, this finding was more pronounced for the 10% NoGo condition [F(1,15) = 7.00, p < .05].

### Reaction Times

The initial comparison between the patient groups and controls examined reaction times (RTs) to correct Go trials only. There were main effects of probability [F(1,32) = 52.52, p < .0001] and group [(F(2,32) = 4.74, p < .05], and an interaction between probability and group [F(2,32) = 3.23, p = .05]. All subjects were faster to respond to targets in the 10% NoGo condition than in the 50% condition. LIFG patients were significantly slower overall than controls (p < .0001), but OFC patients were not (p > .2). The LIFG patients showed a larger difference between RTs in the two probability conditions (69 ms) than controls (40 ms). They were significantly slower than controls in the 50% condition [F(1,26) = 9.84, p < .005], but only marginally so in the 10% condition [F(1,26) = 4.99, p < .06]. There was no difference in RTs between LIFG patients who responded with the left or right hand (p = .26), nor was there an interaction with probability (p = .77). In contrast, the OFC patients did not differ from controls, either in overall speed or in the pattern of RTs in the two probability conditions (p's > .2).

An additional ANOVA compared response times for correct Go trials and incorrect NoGo trials, indicating that all subjects had faster RTs on incorrect NoGo trials than on correct Go trials, suggesting that impulsive responding led to the majority of errors in performance (Fig. 3, bottom). This effect was indicated by a main effect of accuracy [F(1,32)= 68.69, p < .0001] that did not interact with probability (p > .9) or group (p > .3).

### Activation Likelihood Estimation

The map produced by the ALE meta-analysis identified the regions of activation common to successful response inhibition in all 39 studies (Fig. 2). Thirteen clusters were identified, with the largest being centered in the right middle frontal gyrus (BA 9, 46) and insular cortex (BA 13), superior frontal gyrus (medial BA 6), and right inferior parietal lobule/precuneus (BA 40, 7). Also notable is a large cluster in the left insula that extends into the putamen, which overlaps with the insular region damaged in the LIFG patient group (compare Figs. 1 and 2). Additional file 4 illustrates the LIFG lesion overlap and the ALE map on axial slices at the same orientation.

## Discussion

The present study demonstrated that the LIFG is critical for suppressing prepotent responses to simple letter stimuli in a Go/NoGo task. Patients with lateral PFC lesions that included left posterior IFG and frontal opercular regions made more false alarm errors than controls, particularly when response inhibition was more difficult. Conversely, similar to controls, they showed faster RTs on error trials than on correct trials and a low rate of misses, suggesting that impulsive responding, rather than a failure to comply with task instructions or to maintain task set, can account for the increase in NoGo errors. This deficit in response inhibition was not initially predicted by the neuroimaging literature, which has focused almost exclusively on RIFG. The meta-analysis shown in Fig. 2 (see also [40-42]) and numerous fMRI experiments [3] suggest that activation in right inferior and middle frontal gyri is associated with response inhibition to a greater extent than the corresponding left hemisphere regions. Our results provide a relatively unique example of how neuropsychological data can constrain models of cognitive function developed mainly from fMRI data.

An intriguing new possibility, however, also emerged from our ALE map, which is the most comprehensive meta-analysis of motor response inhibition tasks to date: the importance of bilateral anterior insular regions in Go/NoGo and Stop-Signal tasks. Wager, Nee, and colleagues have made this observation as well, based on prior experimental [25] and meta-analytic [43] evidence from tasks of interference resolution. Although a common finding in neuroimaging studies, the importance of the insula in response inhibition has not been widely discussed in the literature, nor has it been reported in previous lesion studies. The maximal overlap in the current patient group includes portions of both left IFG (especially pars opercularis and rolandic regions) and left insula, so we cannot distinguish the relative contributions of each.

Nonetheless, the current lesion study has yielded a deficit in patients that was not generally predicted by the neuroimaging literature. While the present study does not question the importance of RIFG in response inhibition, it does draw attention to the complementary nature of results from neuropsychology and neuroimaging [44-46] and shows that functional imaging results should not become the sole source for generating hypotheses in cognitive neuroscience. Shallice [47] has noted some of the pitfalls of comparing lesion and neuroimaging results, particularly for cognitive processes that are poorly understood. Another caveat is that the degree of lateralization in neuroimaging studies is often relative and not absolute. Thus, a unilateral lesion may not produce a deficit predicted by the neuroimaging data [45] if the spared hemisphere can compensate (or vice versa). Our results suggest that current imaging techniques may not identify every brain area that makes a significant contribution to a particular function, although the possibility of type II error ("false negatives") in fMRI analyses cannot be overlooked.

Aron and Poldrack [48] have argued that response inhibition is right lateralized, which receives support from the quantitative meta-analysis presented in Fig. 2. A model of fronto-striatal loops implementing motor inhibition is quite plausible [48], but it does not explain why fronto-striatal loops of the right hemisphere are dominant for inhibitory control. The RIFG does not appear to have privileged access to the indirect fronto-striatal pathway and is not likely to have direct projections to motor cortices. Thus, no specific anatomical asymmetries between left and right inferior frontal cortices can account for why LIFG should not play a role in inhibitory control at all. So, extant anatomical knowledge alone would probably not hint at a unique role for RIFG in inhibitory control.

The clear performance deficit in LIFG patients suggests that response inhibition processes are represented bilaterally in IFG. Nevertheless, our results do not preclude the possibility that RIFG patients would be even more impaired on this task. However, the contribution of LIFG to inhibitory control is more than minor, since the spared RIFG was not sufficient to compensate for the effect of the LIFG lesion. Previous studies included fewer left unilateral PFC patients than the current experiment, and did not employ direct comparisons between patient groups [27,29]. One of these studies did not find a correlation between Stop-Signal RT and LIFG lesions [27,49] and the other found a correlation with lesions in left BA 6 [29]. The lesion locations in individual patients were not presented in these papers, complicating a direct comparison to the present results.

Another potential explanation for this discrepancy is that different tasks were used. Are there cognitive and/or motor differences between Stop-Signal (SSRT) and Go/NoGo (GNG) tasks that would recruit different regions (or different hemispheres) in PFC? In general, the extent and laterality of IFG activations reported in neuroimaging studies do not differ between the two tasks. Only two studies have administered GNG and SSRT to the same groups of subjects. Zheng et al. [50] implicated right middle frontal gyrus as a key region in both tasks. However, Rubia and colleagues [22] reported that although overlapping PFC regions were activated in GNG and SSRT tasks, the former had more L hemisphere involvement, the latter more R hemisphere involvement.

Very recently, some theorists have proposed that the Go/NoGo task and the Stop-Signal task measure different aspects of response inhibition (Aron and Poldrack [48]; Eagle et al. [51]). Eagle, Bari, and Robbins [51] divided "action inhibition" into different subtypes with distinct neuroanatomical and psychopharmacological correlates. Following Schachar et al. [52], they distinguished between action restraint – inhibition of a motor response *before *the response has been started, and action cancellation – inhibition of a motor response *during *its execution. This model of response inhibition views the Go/NoGo task as an example of action restraint, whereas the Stop-Signal task is an example of action cancellation. Furthermore, the GNG task is thought to be dependent on serotonin (SSRT is not), while SSRT might be dependent on norepinephrine, although this latter point was not entirely clear [51]. The GNG task also seems to be influenced by norepinephrine, implying that the two tasks share some of the same neural substrates.

The paper by Schachar et al. [52] is notable here, because it is the first to test the same group of subjects on the standard SSRT (cancellation) and a new version that is similar to GNG (restraint). The participants were children with and without ADHD. Interestingly, performance on the restraint and cancellation variants was significantly correlated in the control children, suggesting that the two tasks assess a common latent inhibition construct and share cognitive and neural resources. Furthermore, children with ADHD were impaired in both versions of the task, and their performance did not show a correlation between the two tasks, suggesting less sharing of resources in ADHD [53].

Robertson and colleagues [54] have argued that in addition to motor response inhibition, the Go/NoGo task is a measure of sustained attention. Both motor response inhibition and/or sustained attention deficits can produce high NoGo error rates. Two versions of the Sustained Attention to Response Task (SART), a variant of the Go/NoGo task, were developed to target this ambiguity [54,55]. In the random SART, subjects withhold responses to the digit "3" (randomly intermixed with other digits 11% of the time), but in the fixed SART, the numbers always proceed in numerical order. In the random SART, lapses of attention, perseveration, and failures of inhibition can all lead to false alarm errors, whereas with completely predictable NoGo trials in the fixed SART, false alarms are primarily due to lapses of attention. TBI patients were impaired in both, but disproportionately so in the fixed SART [54].

In our experiment, the 10% NoGo blocks might be comparatively more monotonous than the 50% blocks, so sustained attention is required to a greater degree in the former. LIFG patients showed a larger difference between RTs in the two probability conditions than controls. This alone would be consistent with the sustained attention account, in which speeding up in the 90/10 condition can be attributed to entering "autopilot" mode. However, the 10% NoGo condition differs from the fixed SART in that the NoGo stimuli are unpredictable. Importantly, the LIFG patients showed increased false alarm rates in both conditions. Although the percentage of error trials is higher in the 10% condition, the absolute number of errors is similar. Thus, another possibility is that the subjects responded on a small percentage of trials without considering the Go/NoGo signal at all. This type of error was increased in the LIFG group, exemplifying an important form of impulsive responding. Therefore, an inhibitory control deficit remains the best explanation for the LIFG patients' performance.

Further work is required to elucidate the precise nature of response inhibition in both the GNG and the SSRT tasks. For example, there is clear evidence that motor preparation occurs on both Go and NoGo trials [56] so to some extent this task can be considered not only in the light of action restraint, but also as a form of action cancellation. Moreover, recent conclusions based on the SSRT task, with respect to the nature of inhibitory control, may not be definitive at this point. Along these lines, a unique aspect of the SSRT task is that some versions involve switching attention across modalities, from a visual target to an auditory stop-signal. Therefore, alternative interpretations of SSRT results are possible, incorporating both response inhibition processes and the ability to switch attention to the stop-signal tone [57]. Future neuropsychological and neuroimaging studies should test the same groups of subjects on both tasks.

Evidence against a highly specific link between inhibition and RIFG has been accumulating. Impairments in response inhibition have been reported in patients with dorsomedial frontal damage [9-12]. A recent fMRI study associated greater activation in left superior frontal gyrus (BA 8) with more efficient response inhibition [58]. Importantly, a new meta-analysis [59] classified Go/NoGo tasks as either simple (the NoGo stimulus was always the same) or complex (the NoGo stimulus changed depending on context). Common to both task types was greater activation in the pre-supplementary motor area (SMA) during response inhibition (see also Fig. 2), but activation in right dorsolateral PFC was observed only in the complex tasks, which made demands on working memory. As a new theoretical framework incorporating these findings develops, the emerging emphasis is likely to be on a well-circumscribed but anatomically distributed frontal lobe inhibitory control network. A core element in this network includes pre-SMA circuits, with recruitment of additional frontal (and posterior) regions perhaps varying according to task demands [35].

Returning to the idea of a unified hypothesis of LIFG function, a key commonality involves restraining alternatives in a given context that includes motor, semantic, mnemonic, or linguistic alternatives. Semantic selection [30] involves inhibition of unselected alternatives; speech production has both cognitive and motor control components, possibly tapping into general purpose selection/inhibition mechanisms [60]; vocal control for speech might share evolutionary origins with manual motor control for gesturing [61]; left hemisphere dominance for action might have implications for motor response control [62]; rejection of new items in a recognition memory task might involve inhibition of any tendency to generate a yes response [63]. The present data add the inhibition of dominant motor response tendencies to this roster. Another possibility to consider, for posterior LIFG at least, is that subvocalization is actually a critical aspect of many complex cognitive activities, as speculated in a review article on the role of inner speech in self-reflective processing [64]. Although beyond the scope of this particular paper, ongoing research is investigating a parcellation of LIFG cognitive control functions along the anterior-posterior dimension [65].

Interestingly, OFC patients did not commit a greater number of false alarm errors, contradicting a general characterization as impulsive in all behavioral domains. This lends a degree of anatomical specificity to the LIFG inhibitory control impairment. On the other hand, all of the OFC patients suffered head trauma, and this finding diverges from some results in TBI patients [54], but not others [66]. This latter study did not find a deficit in the random SART in a group of 26 TBI patients [66]. While the differences in the time post-injury and differences between standard Go/NoGo and SART procedures may account for the spared performance in OFC patients, the current finding is of theoretical interest in relation to OFC function.

The present findings have significance from a clinical standpoint as well. A number of different psychiatric disorders have been described as dysfunctions of "frontal" inhibitory processes that involve increases in impulsive behavior, motivating investigators to explore which frontal areas might be dysfunctional in various psychiatric conditions. The Go/NoGo task has been used by various researchers to investigate the biological basis of motor impulsiveness [67], mainly relying on neuroimaging and electrophysiological data [68-70]. Human lesion studies with precise neuroanatomical characterization of the PFC regions underlying these different types of disinhibition can contribute to a better understanding of the neurobiological correlates of disorders such as ADHD, alcoholism, drug abuse, schizophrenia, and obsessive-compulsive disorder.

## Conclusion

The present results indicated that patients with lesions in LIFG were impaired at inhibiting motor responses in a GoNoGo task. The deficit occurred when NoGo responses were rare (10%) as well as frequent (50%), but to a greater degree in the former condition, when Go responses were more compelling. This pattern of results could suggest deficits in multiple forms of attentional control required to perform this task. Impairments in the 50% NoGo condition could directly reflect an inability to inhibit responses even when they are not prepotent. Difficulties in the 10% NoGo condition could reflect not only a deficit in response inhibition, but also problems with sustained attention [54] or attentional control processes required to maintain a rule that is applied only 10% of the time. However, in light of the extant literature and current thinking on inhibitory control mechanisms and the Go/NoGo task, the most likely explanation remains a deficit in response inhibition. Our results demonstrate that successful exercise of inhibitory motor control processes does not rely exclusively on the integrity of RIFG or superior medial areas. LIFG is also critical for suppressing prepotent but inappropriate responses.

## Authors' contributions

DS conceived and designed the experiments, performed statistical analyses, and drafted the manuscript. VA performed the experiments and analyzed the data. AT contributed analysis ideas and helped to draft the manuscript. All authors read and approved the final manuscript.

## Supplementary Material

Additional file 1 **Lesion Reconstructions for LIFG Patients**. Lesion reconstructions for the individual patients with lesions of the left inferior frontal gyrus (LIFG). Lesions were estimated from MRI or CT scans and transcribed onto sequential axial templates derived from the MNI brain. Lesions are shown from ventral to dorsal. Click here for file

Additional file 2 **Lesion Reconstructions for OFC Patients**. Lesion reconstructions for the individual patients with lesions of the orbitofrontal cortex (OFC). Lesions were estimated from MRI or CT scans and transcribed onto sequential axial templates derived from the MNI brain. Lesions are shown from ventral to dorsal. Click here for file

Additional file 3 **Full List of References for the ALE Meta-Analysis**. Thirty-nine papers were found to meet the criteria for inclusion in the Activation Likelihood Estimation analysis of response inhibition conditions in Go/NoGo and Stop-Signal tasks: 25 papers were downloaded from the BrainMap database, and 14 were found through PubMed searches. Click here for file

Additional file 4 **Averaged LIFG Lesion Reconstruction and ALE Map**. Both are illustrated on axial templates that are matched for slice angle. (A) Lesion overlap in patients with damage to the left inferior frontal gyrus. (B) Activation likelihood estimation (ALE) map showing significant inhibition-related activations. Click here for file
