# Supplementary figures and images for: Left inferior frontal gyrus is critical for response inhibition
Source: BMC Neurosci. 2008 Oct 21;9:102. doi: 10.1186/1471-2202-9-102 (PMC2588614; doi:10.1186/1471-2202-9-102)

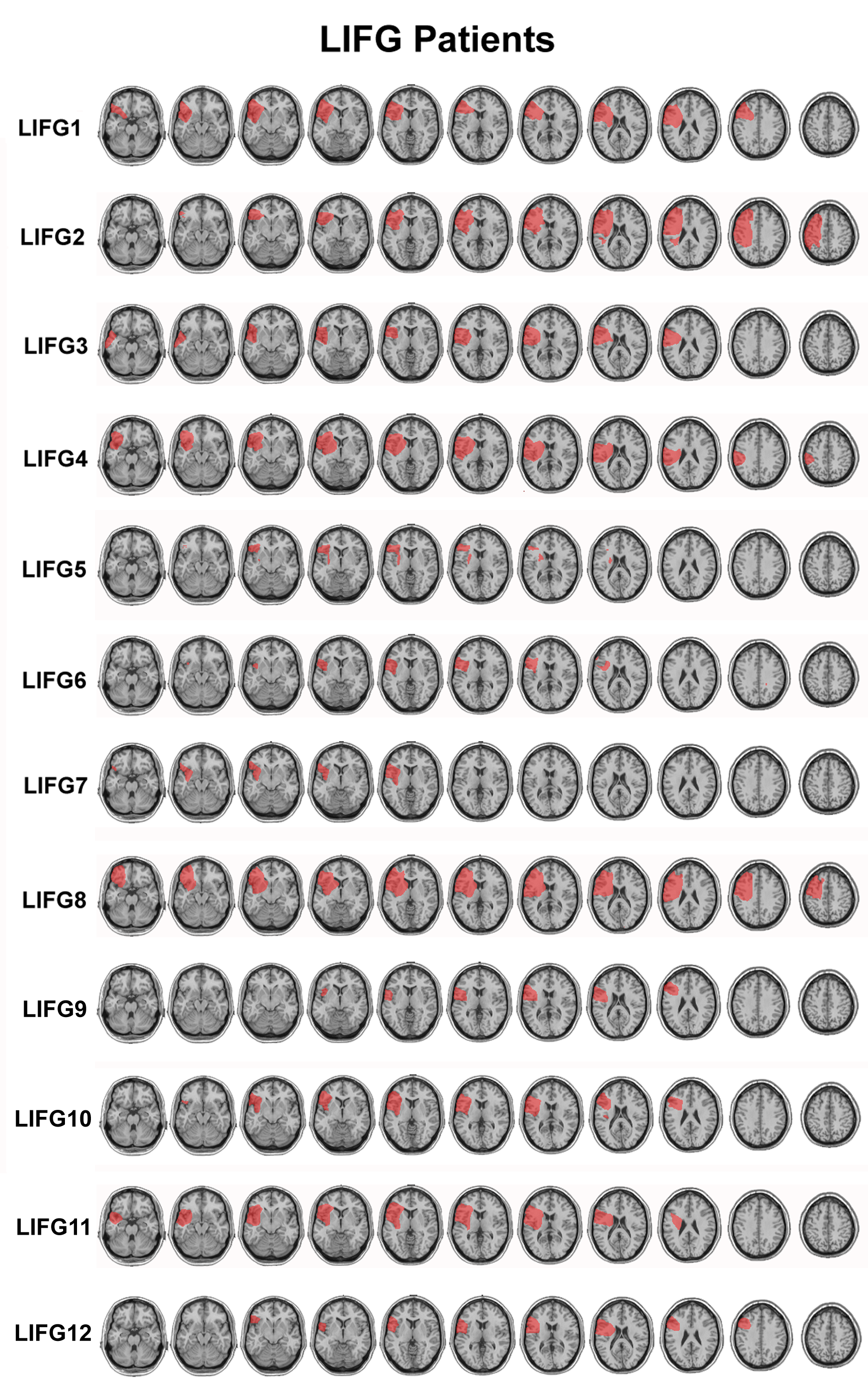

Supplement: Additional file 1 — Lesion Reconstructions for LIFG Patients. Lesion reconstructions for the individual patients with lesions of the left inferior frontal gyrus (LIFG). Lesions were estimated from MRI or CT scans and transcribed onto sequential axial templates derived from the MNI brain. Lesions are shown from ventral to dorsal. [file 1471-2202-9-102-S1.tiff]

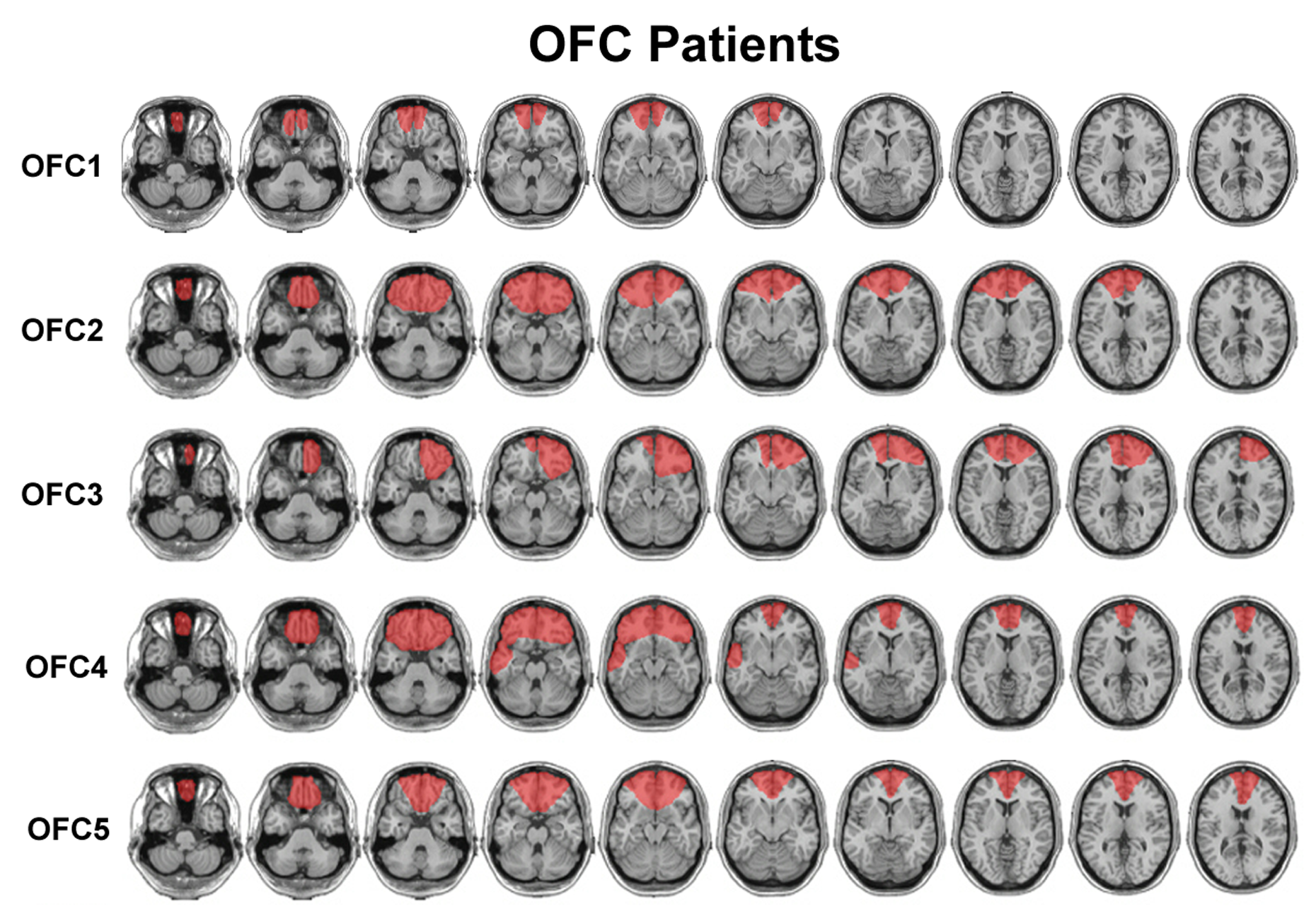

Supplement: Additional file 2 — Lesion Reconstructions for OFC Patients. Lesion reconstructions for the individual patients with lesions of the orbitofrontal cortex (OFC). Lesions were estimated from MRI or CT scans and transcribed onto sequential axial templates derived from the MNI brain. Lesions are shown from ventral to dorsal. [file 1471-2202-9-102-S2.tiff]

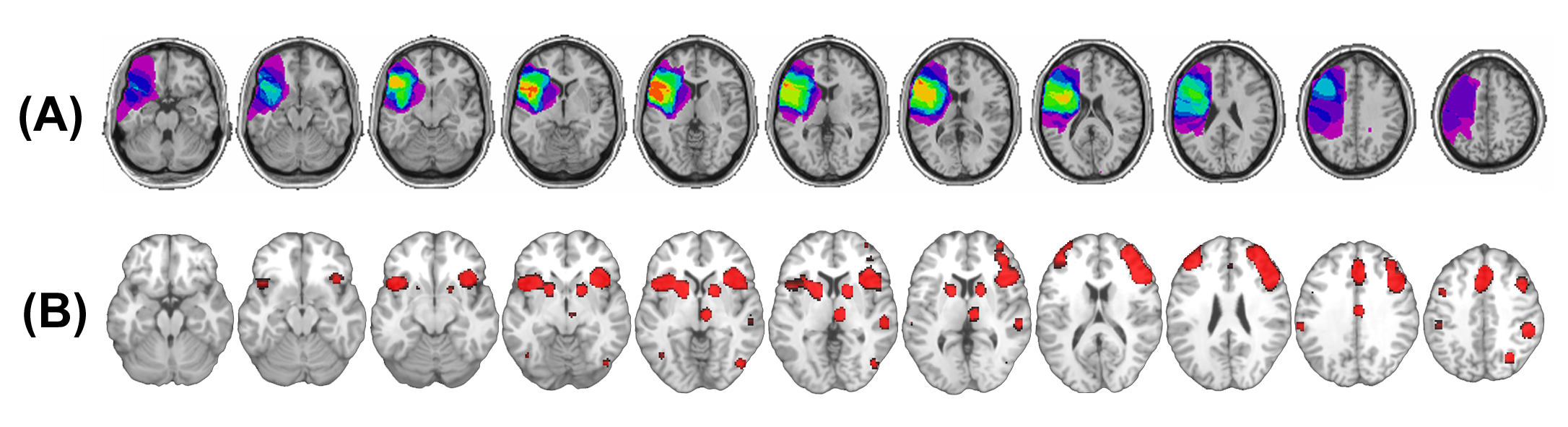

Supplement: Additional file 4 — Averaged LIFG Lesion Reconstruction and ALE Map. Both are illustrated on axial templates that are matched for slice angle. (A) Lesion overlap in patients with damage to the left inferior frontal gyrus. (B) Activation likelihood estimation (ALE) map showing significant inhibition-related activations. [file 1471-2202-9-102-S4.tiff]
