# Supplementary material for: Left inferior frontal gyrus is critical for response inhibition
Source: BMC Neurosci. 2008 Oct 21;9:102. doi: 10.1186/1471-2202-9-102 (PMC2588614; doi:10.1186/1471-2202-9-102)
Supplement: Additional file 3 — Full List of References for the ALE Meta-Analysis. Thirty-nine papers were found to meet the criteria for inclusion in the Activation Likelihood Estimation analysis of response inhibition conditions in Go/NoGo and Stop-Signal tasks: 25 papers were downloaded from the BrainMap database, and 14 were found through PubMed searches. [file 1471-2202-9-102-S3.doc]

# Supplementary References

Aron AR, Behrens TE, Smith S, Frank MJ, Poldrack RA: **Triangulating a cognitive control network using diffusion-weighted magnetic resonance imaging (MRI) and functional MRI**. *J Neurosci* 2007, 27: 3743-3752.

Aron, AR, Poldrack, RA: **Cortical and subcortical contributions to Stop signal response inhibition: role of the subthalamic nucleus.** *J Neurosci* 2006, 26: 2424-2433.

Asahi S, Okamoto Y, Okada G, Yamawaki S, Yokota N: **Negative correlation between right**

**prefrontal activity during response inhibition and impulsiveness: a fMRI study.** *Eur*

*Arch Psychiatry Clin Neurosci* 2004, 254: 245-251.

Bellgrove MA, Hester R, Garavan H: **The functional neuroanatomical correlates of response**

**variability: evidence from a response inhibition task.** *Neuropsychologia* 2004, 42:

1910-1916.

Braver TS, Barch DM, Gray JR, Molfese DL, Snyder A: **Anterior cingulate cortex and**

**response conflict: effects of frequency, inhibition and errors.** *Cereb Cortex* 2001, 11: 825-836.

Chikazoe J, Jimura K, Asari T, Yamashita K-I, Morimoto H, Hirose S, Miyashita Y, Konishi S:

**Functional Dissociation in Right Inferior Frontal Cortex during Performance of Go/No-Go Task.** *Cereb Cortex* 2008, Apr 28 [Epub ahead of print].

de Zubicaray GI, Andrew C, Zelaya FO, Williams SC, Dumanoir C: **Motor response suppression and the prepotent tendency to respond: a parametric fMRI study.**

*Neuropsychologia* 2000, 38: 1280-1291.

Fassbender C, Murphy K, Foxe JJ, Wylie GR, Javitt DC, Robertson IH, Garavan H: **A**

**topography of executive functions and their interactions revealed by functional**

**magnetic resonance imaging.** *Cog Brain Res* 2004, 20:132-143.

Garavan H, Ross TJ, Stein EA: **A Right hemispheric dominance of inhibitory control: an event-related functional MRI study.** *Proc Natl Acad Sci* 1999, 96: 8301-8306.

Garavan H, Ross TJ, Murphy K, Roche RA, Stein EA: **Dissociable executive functions in the**

**dynamic control of behavior: inhibition, error detection, and correction.** *Neuroimage*

2002, 17: 1820-1829.

Garavan H, Ross TJ, Kaufman J, Stein EA: **A midline dissociation between error processing**

**and response-conflict monitoring.** *Neuroimage* 2003, 20: 1132-1139.

Hester RL, Murphy K, Foxe JJ, Foxe DM, Javitt DC, Garavan H: **Predicting success: patterns**

**of cortical activation and deactivation prior to response inhibition.** *J Cog Neurosci*

2004, 16: 776-785.

Horn NR, Dolan M, Elliott R, Deakin JF, Woodruff PW: **Response inhibition and impulsivity:**

**an fMRI study.** *Neuropsychologia* 2003, 41: 1959-1966.

Kaladjian A, Jeanningros R, Azorin JM, Grimault S, Anton JL, Mazzola-Pomietto P: **Blunted**

**activation in right ventrolateral prefrontal cortex during motor response inhibition**

**in schizophrenia.** *Schizophrenia Res* 2007*,* 97: 184–193.

Karch S, Jäger L, Karamatskos E, Graz C, Stammel A, Flatz W, Lutz J, Holtschmidt-Täschner B,

Genius J, Leicht G, Pogarell O, Born C, Möller HJ, Hegerl U, Reiser M, Soyka M,

Mulert C: **Influence of trait anxiety on inhibitory control in alcohol-dependent patients: Simultaneous acquisition of ERPs and BOLD responses.** *Journal of Psychiatric Research* 2008, 42: 734–745.

Kawashima R, Satoh K, Itoh H, Ono S, Furumoto S, Gotoh R, Koyama M, Yoshioka S,

Takahashi T, Takahashi K, Yanagisawa T, Fukuda H: **Functional anatomy of GO/NO-**

**GO discrimination and response selection--a PET study in man.** *Brain Res* 1996, 728:

79-89.

Kelly AM, Hester R, Murphy K, Javitt DC, Foxe JJ, Garavan H: **Prefrontal-subcortical**

**dissociations underlying inhibitory control revealed by event-related fMRI.** *Eur J*

*Neurosci* 2004, 19: 3105-3112.

Kiehl KA, Liddle PF, Hopfinger JB: **Error processing and the rostral anterior cingulate: an**

**event-related fMRI study.** *Psychophysiol* 2000, 37: 216-223.

Konishi S, Nakajima K, Uchida I, Sekihara K, Miyashita Y: **No-go dominant brain activity in**

**human inferior prefrontal cortex revealed by functional magnetic resonance**

**imaging.** *Eur J Neurosci* 1998, 10: 1209-1213.

Konishi S, Nakajima K, Uchida I, Kikyo H, Kameyama M, Miyashita Y: **Common inhibitory**

**mechanism in human inferior prefrontal cortex revealed by event-related functional**

**MRI**. *Brain* 1999, 122: 981-991.

Langenecker SA, Kennedy SE, Guidotti LM, Briceno EM, Own LS, Hooven T, Young EA, Akil

H, Noll DC, Zubieta JK: **Frontal and limbic activation during inhibitory control**

**predicts treatment response in major depressive disorder.** *Biol Psychiatry* 2007,

62:1272-80.

Laurens KR, Kiehl KA, Liddle PF: **A supramodal limbic-paralimbic-neocortical network**

**supports goal-directed stimulus processing.** *Hum Brain Mapp* 2005, 24: 35-49.

Leung HC, Cai W: **Common and Differential Ventrolateral Prefrontal Activity during**

**Inhibition of Hand and Eye Movements.** *J Neurosci* 2007, 27: 9893-9900.

Li CS, Huang C, Constable RT, Sinha R: **Gender differences in the neural correlates of**

**response inhibition during a stop signal task.** *NeuroImage* 2006, 32: 1918–1929.

Liddle PF, Kiehl KA, Smith AM: **Event-related fMRI study of response inhibition.** *Hum*

*Brain Mapp* 2001, 12: 100-109.

Maguire RP, Broerse A, de Jong BM, Cornelissen FW, Meiners LC, Leenders KL, den Boer JA:

**Evidence of enhancement of spatial attention during inhibition of a visuo-motor**

**response.** *NeuroImage* 2003, 20: 1339-1345.

Maltby N, Tolin DF, Worhunsky P, O'Keefe TM, Kiehl KA: **Dysfunctional action monitoring**

**hyperactivates frontal-striatal circuits in obsessive-compulsive disorder: an event**

**related fMRI study**. *Neuroimage* 2005, 24: 495-503.

Menon V, Adleman NE, White CD, Glover GH, Reiss AL: **Error-related brain activation**

**during a Go/NoGo response inhibition task.** *Hum Brain Mapp* 2001, 12: 131-143.

Mobbs D, Eckert MA, Mills D, Korenberg J, Bellugi U, Galaburda AM, Reiss AL:

**Frontostriatal dysfunction during response inhibition in Williams syndrome.** *Biol*

*Psychiatry* 2007, 62 :256-261.

Mostofsky SH, Schafer JG, Abrams MT, Goldberg MC, Flower AA, Boyce A, Courtney SM, Calhoun VD, Kraut MA, Denckla MB, Pekar JJ: **fMRI evidence that the neural basis**

**of response inhibition is task-dependent.** *Cog. Brain Res* 2003, 17: 419-430.

Nakata H, Sakamoto K, Ferretti A, Gianni Perrucci M, Del Gratta C, Kakigi R, Luca Romani G:

**Somato-motor inhibitory processing in humans: an event-related functional MRI**

**study.** *Neuroimage* 2008, 39:1858-1866.

Roth RM, Saykin AJ, Flashman LA, Pixley HS, West JD, Mamourian AC: **Event-related**

**functional magnetic resonance imaging of response inhibition in obsessive**

**compulsive disorder.** *Biol Psychiatry* 2007, 62: 901-909.

Rubia K, Russell T, Overmeyer S, Brammer MJ, Bullmore ET, Sharma T, Simmons A, Williams

SC, Giampietro V, Andrew CM, Taylor E: **Mapping motor inhibition: conjunctive**

**brain activations across different versions of go/no-go and stop tasks**. *Neuroimage* 2001, 13: 250-261.

Rubia K, Smith AB, Woolley J, Nosarti C, Heyman I, Taylor E, Brammer M: **Progressive**

**increase of frontostriatal brain activation from childhood to adulthood during**

**event-related tasks of cognitive control.** *Hum Brain Mapp* 2006, 27: 973-993.

Vink M, Kahn RS, Raemaekers M, van den Heuvel M, Boersma M, Ramsey NF: **Function of**

**striatum beyond inhibition and execution of motor responses.** *Hum Brain Mapp* 2005,

25: 336-344.

Wager TD, Sylvester CY, Lacey SC, Nee DE, Franklin M, Jonides J: **Common and unique**

**components of response inhibition revealed by fMRI**. *Neuroimage* 2005, 27: 323-340.

Watanabe J, Sugiura M, Sato K, Sato Y, Maeda Y, Matsue Y, Fukuda H, Kawashima R: **The**

**human prefrontal and parietal association cortices are involved in NO-GO**

**performances: an event-related fMRI study.** *Neuroimage* 2002, 17: 1207-1216.

Xue G, Aron AR, Poldrack RA: **Common Neural Substrates for Inhibition of Spoken and**

**Manual Responses**. *Cereb Cortex* 2008, 18: 1923-1932.

Zheng D, Oka T, Bokura H, Yamaguchi S: **The Key Locus of Common Response Inhibition**

**Network for No-go and Stop Signals.** *J Cogn Neurosci* 2008, 20: 1434-1342.
